# Women´s experiences of preeclampsia as a condition of uncertainty: a qualitative study

**DOI:** 10.1186/s12884-022-04826-5

**Published:** 2022-06-28

**Authors:** Therése Hansson, Maria E. Andersson, Gerd Ahlström, Stefan R. Hansson

**Affiliations:** 1 Institution of Clinical Sciences Lund, Lund University, Lund, Sweden; 2 Ystad Hospital, Ystad, Sweden; 3 Department of Obstetrics and Gynecology, Institution of Clinical Sciences Lund, Lund University, Lund, Sweden; 4 Skane University Hospital (SUS), Malmö/Lund, Sweden; 5 Department of Health Sciences, Faculty of Medicine, Lund University, Lund, Sweden

**Keywords:** Preeclampsia, Experience, Qualitative research, In-depth patient interviews, Phenomenology, Outcomes

## Abstract

**Background:**

Preeclampsia is a severe condition that annually affects about 3–8% of pregnancies worldwide. Preeclampsia is thereby one of the most common pregnancy complications for both mother and child. Despite that, there is limited research exploring the women´s perspective of experiencing preeclampsia.

**Aim:**

The aim of this study was to describe women´s experiences of preeclampsia to improve the support and care given during and after pregnancy.

**Methods:**

A qualitative descriptive interview study was undertaken. Nine women, diagnosed with preeclampsia, were recruited from a maternity unit in southern Sweden. The descriptive phenomenological method according to Amadeo Giorgi was used to analyse the data.

**Results:**

The women´s experiences of PE were expressed as A condition of uncertainty, meaning that it was an unexpected and unknown situation. This main result consisted of 1) incomprehensible diagnosis message, 2) ambivalent feeling when the unexpected happens, 3) confusing contradictory messages, 4) appreciated support from the midwife, 5) need for continuous information. The nature of preeclampsia can sometimes deteriorate rapidly both for the mother and/or the child, often resulting in conversion from a planned vaginal spontaneous delivery to an emergency Caesarean section. The women narrated diffuse symptoms, and they experienced that they got contradictory information from different health care professionals regarding the severity of their disease. Detailed and continuous information is requested throughout the course of the disease, and the postpartum period.

**Conclusion:**

This qualitative study reveal a need for improved clinical management. Health care professionals must be aware that women and their partners need detailed, consistent and repeated information about severity and prognosis to diminish the condition of uncertainty, confusion and fearful experience. The clinical implication would be a standardized preeclampsia education for pregnant women early on in the pregnancy, to raise awareness of preeclamptic symptoms. Furthermore, there is a need for harmonized guidelines and individualized support to the woman and her partner both at the antenatal care and the maternity ward and inpatient care at the hospital.

## Introduction

Preeclampsia (PE) is one of the most common severe pregnancy complications for both mother and child [1], a condition that affects about 3.5% pregnant women in Sweden [2] annually, and about 3–8% of all pregnant women worldwide [1]. Preeclampsia contributes to high maternal (18%) and fetal (40%) mortality globally [1, 3]. The cause of PE is partly unknown but inadequate placentation and or placenta dysfunction plays a central role [4]. Uneven blood perfusion results in oxidative stress and placenta damage [5]. This in turn causes leakage of fetal cells, placenta debris and microparticles into the maternal circulation, which give rise to inflammation and general endothelium damage, a hallmark of PE [6]. Endothelial damage is believed to be the underlying cause for organ dysfunction and specific manifestations from the kidneys, liver, heart, lungs, clotting system and the brain [1, 7]. The disturbed placenta function in PE also affects the fetus and causes fetal growth restrictions (FGR) in 25% of the cases and 15% of all premature birth [7, 8].

The definition of PE, is high blood pressure, ≥ 140 mmHg systolic blood pressure (sBP) and > 90 mmHg diastolic blood pressure (dBP), after the 20^th^ gestation week, combined with organ engagement including FGR [9]. Early-onset PE is defined as delivery due to PE before 34 weeks of pregnancy [7, 9].

Treatment of PE is largely symptomatic, with antihypertensive drugs to manage the blood pressure, magnesium sulphate to prevent and treat eclampsia and steroids for the fetal lung maturation. To date, the most definitive treatment is delivery and removal of the placenta, which generally stops progression of the disorder. However, this is a decision that can be difficult since it has consequences for both mother and child [1, 7]. International guidelines recommend induction of delivery no later than 37 gestational weeks in women diagnosed with preeclampsia [10].

Preeclampsia is associated with long-term sequelae for the mothers. There is evidence that there is an increased risk for PE women to develop hypertension, stroke, type II diabetes and cardiovascular disease later in life [11], but also for depression and posttraumatic stress syndrome [12, 13].

In qualitative studies, women often describe PE as a frightening and life-threatening condition, with lack of care and low psychosocial support, experiences that often collides with their expectations of pregnancy and childbirth [14, 15]. Instead, they experience anxiety, associated with hospitalization, multiple controls, emergency Caesarean sections (CS), premature babies and separation from their child [16]. Studies show that women with a complicated childbirth where the baby needs treatment at the Neonatal Intensive Care Unit (NICU) find it to be a traumatizing experience to be separated from their child [16, 17].

There is a need for continuous information about their own health, and especially about their child’s wellbeing. Family centred care is recommended for social support and for development of the parent–child relationship after pregnancy complications such as PE [16, 18].

A recent narrative synthesis, based on 10 studies, showed a lack of knowledge and understanding about PE as a disease among women and their families [19]. Some PE women did not experience any symptoms at all, or not the “classical” ones, which led to delayed diagnosis of early-onset PE [19]. Suffering from PE is very stressful and the women also experience fragmented care and information [19]. These studies indicate that there is a great need for additional support, patient information and education regarding PE among pregnant women [17–19]. It is also important to get the women to attend their antenatal routine care [19], something that is highlighted in several studies [18, 20, 21].

Despite the fact that Sweden has a well-developed maternal health care system, free of charge and with good accessibility, there is still a lack of knowledge regarding PE among pregnant women.

Worldwide, few studies describe women´s experiences of having PE. By using the descriptive phenomenological method the knowledge gap could be diminished when the focus is centered on the women’s voices and thereby new information and nuances can appear, that otherwise could be lost. The aim of this study was therefore to describe women´s experiences of PE in order to improve support and care given during, as well as after a pregnancy complicated by PE.

## Methods

### Design and research perspective

A qualitative descriptive interview study was performed. The data was analysed using a phenomenological approach of psychological and human science according to Amadeo Giorgi [22–24]. Phenomenology aims to describe our experience of the world as it appears, before all theories and criticisms. The researcher puts the world in brackets, to peel away his/her preconceptions and beliefs about how the world works, also called phenomenological reduction [20]. The theoretical understanding develops in the light of understanding the lifeworld, which is the sum of physical surroundings and experiences that make up an individual’s world. According to phenomenology, to gain knowledge, the lifeworld precedes the theory and should be studied first and foremost [25, 26].

### Participants

The selection of a purposeful sample was carried out at a maternity unit in southern Sweden. The unit has approximately 5500 deliveries annually and the PE incidence is about 3.6% [2]. The inclusion criteria were diagnosed with PE, delivered within 5 days, > 18 years, understanding and speaking Swedish. For a period of three months, the admitted women that met the inclusion criteria and accepted participation in the study were included.

Exclusion criteria were other pregnancy complications and fetal/infant death. The exclusion criteria were chosen to focus on and refine the PE experience. Situations with fetal/ infant death are associated to PE but still rare. Those situations are such a trauma for the women that we found it unethical to burden them with a study.

In total nine (*n* = 9) newly delivered PE women were included.

Table 1 describes the characteristics of the participants with PE, and complicating factors, mode of delivery and hospitalization time. Seven out of nine women were delivered by CS, only one during general anaesthesia. Three women presented with early onset PE, and seven out of nine babies were SGA (two IUGR after correlated with birth weight). After CS, the women spent their first hours in the post-operative unit, separated from their children, who were with their partners or in NICU (Table 1).

**Table 1:** Characteristics of women with preeclampsia in the study (*N* = 9)

**Characteristics**	
Age (years), mean ± SD	30.4 ± 10.6
BMI, mean ± SD	25.8 ± 6.8
Primigravity, n (%)	2 (22%)
**Medical condition, n (%):**	
Family high bloodpressure	3 (33%)
IVF	2 (22%)
Astma	2 (22%)
Depression	1 (11%)
DVT, APC resistens	2 (22%)
**Gestational age,** mean ± SD:	
Preterm birth (< 37 weeks)	4 (31 + 0—36 + 0)
Term birth (> 37 weeks)	5 (37 + 1—38 + 3)
**Gestational Weight, g (mean):**	1500-3115 g (mean 2060 g)
**Mode of delivery, n (%):**	
Vaginal	2 (22%)
Caesarean section	7 (77%)
**Newborn in NICU, n (%):**	5 (56%)
**Days in hospital care:**	
Antenatal	1–14 days
Postnatal	3–7 days

*BMI* body mass index, *DVT* Deep Vein Thrombosis, *NICU* Neonatal Intensive Care Unit, *IVF* In Vitro Fertilisation, *APC* Activated Protein C Resistance

### Data collection

The included women received oral and written information regarding the study, and gave their signed informed consent based on voluntary decisions. The interviews were performed in a private room at the maternity unit, or in a private part of the NICU, and were recorded digitally. In three of the nine interviews, the fathers attended.

The interviews consisted of one opened-ended question, which allowed the women to freely speak about their experiences and their lifeworld, without leading their answers into a given direction. All the women received this opening question: -Would you like to tell me about you experiences of living with PE? In some cases, there were a clarifying follow-up question, in order to explain the answer, e.g.:—“Can you tell me more about how you were feeling? Can you say something more about that?” The researcher assumed the phenomenological attitude, to sit back and be like an open book, and lay aside all preconceptions to let the woman speak about her experiences [24]. The interviews lasted between 25–60 min and were transcribed verbatim.

### Data analysis

The analyses were conducted according to Giorgi´s descriptive phenomenological method [22, 27], consisting of a five steps structure, to gain insight about the constituents that are parts of and generates the essence of the phenomenon.

The five steps in the analysis were performed by the first author (TH) with bracketing of the preconceptions [24]: 1. The verbatim transcribed interviews were read thoroughly to obtain an overall impression; 2. Unique meaning units were identified in the transcribed text, and these units consist of one or more sentences or paragraphs i.e. a new meaning unit for each new content of the text; 3. The meaning units were reflected upon and then thematised based on the women´s point of view; 4. The thematised meaning units were further concentrated to produce the essential significance units; 5. In the last step, constituents were emerging, which are the parts that generates the essential structure of the phenomenon [24]. When all nine interviews were analysed according to these five steps, several common/united constituents were revealed, merged to the essence and the phenomenon appeared [28, 29]. After the analysis, the essence and the constituents were reviewed and discussed with two researchers (G.A, M.A).

## Results

**Fig. 1:**
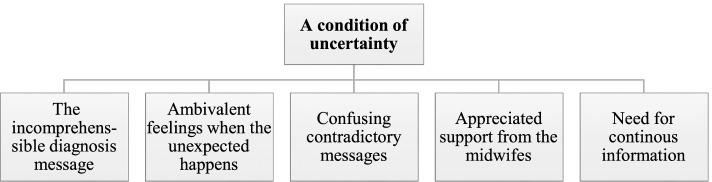
The essence and the five constituents

Being diagnosed with PE, and having a pregnancy affected by possible complications and outcomes, leads to the main essence of the phenomenon of women´s experiences of PE, described as “a condition of uncertainty”*.* Women found themselves posing more questions than they got answers, trying to grasp the complexity of their PE diagnosis, for both themselves and their unborn baby. PE was described as an unexpected, uncertain, and unknown condition. The essence is generated from five identified constituents, described with representative quotes (Fig. 1).

### The incomprehensible diagnosis message

Several of the women in the study had little knowledge of PE, and some women expressed that they did not know it was a serious condition. They expressed that the PE-symptoms were diffuse, and hard to distinguish from normal pregnancy symptoms; this was especially problematic for nulliparous mothers. Women experiencing the PE diagnosis as incomprehensible stated that they were feeling great and that they did not notice anything different during their pregnancy. Their PE was only detected by blood pressure and proteinuria measurements. These women expressed having problems accepting the diagnosis, the extra controls and sometimes the need of admission to the hospital. The pregnant women wished there was a specific diagnostic test for PE to confirm their diagnosis and the severity of the disease.

*“-But it´s not dangerous to have PE, but it´s not meant that you should walk around with it. So I´m just glad that she (the midwife) discovered it.”* (Interviewee number 7, 29 years, nulliparous).

The fathers that were present during the interviews, at the maternity unit in the hospital or the NICU, affirmed the women’s experiences and concerns. Together the parents tried to grasp what happened.

*“- I think it´s scary that I didn´t have any symptoms”. Father:- “And there were no alarm bells when you didn´t have the usual symptoms (of PE), like blurry vision and headaches”. Woman:- “And it´s the same father for both of my children”. Father:—“It´s scary that it can be a silent disease. In a way it´s like a tumour, you don´t see that it´s growing, but it is”. Woman:—“Yes and then BOOM, it´s just there, and I had to be hospitalized”.* (Interviewee number 9, 29 years, two parous).

The women described a scary feeling of central chest pain/sternum before their condition worsened. They also talked about a diffuse feeling that something was wrong.

*“I just felt, I can´t breathe! It felt just like someone was standing on my chest, and pressed, like with a shoe, really hard. I almost panicked! And I also felt that something is wrong, but it´s just the breathing that I can pinpoint.* (Interviewee number 9, 29 years, two parous).

The PE-diagnosis was not something they expected, and it can be a fearful experience. The women expressed that they did not know how to address the diagnosis, what to do with the information. They reacted on the Swedish word for PE, which sounds alarming, “pregnancy toxication”. It was also a shock for some of the women to comprehend that she was diagnosed with PE.

*“- I was frightened when I got PE. I had all the symptoms, it was only that I didn´t have the proteinuria. When they said that I had protein, I knew, and I thought that now everything can happen. I can die and my baby can die, we can die both. But I thought that I was in the hospital, so it´s a good chance”.* (Interviewee number 2, 34 years, two parous).

### Ambivalent feelings when the unexpected happens

Women expressed ambivalent feelings, both fear and relief, when their PE-condition deteriorated rapidly, and sometimes resulted in conversion from a planned vaginal spontaneous delivery to an emergency CS. The women often told about their birth experience that differed from what they had expected. They experienced a sudden decline in their own or their babies’ wellbeing, which lead to decisions to convert from a planned vaginal delivery to an emergency CS. These unpredictable births were very traumatic, especially if the mothers were anesthetised. The women also expressed a need to talk to somebody about their traumatic experiences, and one woman had already talked twice to the counsellor at the maternity unit.

*“-I had very intense birth pains, but I didn´t open. So the doctor then decided that it was going to be a planned Caesarean section, within an hour. Then I was rolled up (into the operation room), and they prepared me, everything became extremely acute and I was sedated. And then, ….// I think it was her (the baby) heart sounds, that; she didn´t feel well at all.// We had planned that my husband would be beside me, and we would both see her when she.., but then something happened, I don´t know what, and I was sedated and he had to go out, and he didn´t see anything until they had picked her out. It was very daunting.”* (Interviewee number 8, 25 years, nulliparous).

Women with prolonged labour or women who experienced a rapid deterioration in their health status, said that they were relieved and welcomed a decision to convert the mode of delivery to a CS. In addition, women with a previous experience of a pregnancy complicated with PE welcomed a planned CS, even if the baby was slightly premature.

*“Oh, I was so relived! Because I felt that I would not…, and just because she (the baby) was so weak, she might not have pressed herself down, so. I was very relieved!* (Interviewee number 6, 29 years, nulliparous).

### Confusing contradictory messages

The women in the study described that they got confused about the contradictory and different messages from different health care professionals, regarding the severity of their PE. The women expressed that this lack of consensus between antenatal- and inpatient care regarding the PE severity, made them worried and confused whether or not to seek care, and left them in a condition of uncertainty.

*“-What I have experienced here is that when I went to the hospital for controls, they were not worried at all, they had higher barriers to what was alarming compared to the MHC, so it’s a bit confusing actually. I have not been able to figure out what is what, so I think that they can´t hold me responsible for it. Instead of just taking a test and say that it is PE, or not”.* (Interviewee number 1, 27 years, nulliparous).

After being admitted to the hospital, the women received contradictory messages from doctors and midwives regarding their condition. This inconsistency in information regarding their own and their babies´ condition was very disturbing for the women.

*“- And then you meet different doctors every day, new messages all the time, because everybody had different experiences. Yes, because, some said, mostly midwifes and nurses, well, if we only get your blood pressure down and stable, I´m sure you can be discharged and go home. And one doctor said, you will leave with the baby on the “outside”. So it was very contradictory, that is, whom should I listen to in this. If everyone can just agree, so I know how to relate to my condition”* (Interviewee number 9, 29 years, two parous).

### Appreciated support from the midwives

The midwives at the Maternal Health Care (MHC) were greatly appreciated by the women, for their support and care. The women in this study said that their midwife was the first health care professional that informed them of possible PE diagnosis and that sent them to the hospital or made an appointment to the primary health care physician. They appreciated the midwives for taking their condition seriously and their motivating conversations to get them to continue coming for tests and surveys.

*“-They knew that I had a problem with the placenta, and then it got a little bit serious, and she (the midwife) listened very carefully to what I told her about my symptoms. And she told me, you have to come, you have to come, so that it doesn´t becomes a bigger problem”* (Interviewee number 2, 34 years, who previously lost a baby due to PE).

The women were grateful for the midwives´ competent guidance during more investigations and follow-ups.

*“When my blood pressure got high, my midwife at the MHC told me about PE, and that I would try to go on sick leave. Because stress, after all, is never a good combination. So that I still think that they took it really seriously at the check-up, I was sent straight from there to the specialist maternity ward for the first time. The midwife said, “you are not going to go to work today, you are going to go there and do this, and pack your stuff and your papers and prepare because I cannot say what they will answer. So it felt like there was a seriousness here at the MHC.”* (Interviewee number 9, 29 years, two parous).

### Need for continuous information

The women stated that they required detailed, consistent and continuous information, throughout the course of the disease and in the postpartum period. Being diagnosed with PE and all its complications that follows is very concerning for the woman. They all expressed a need for more information, to help them in a condition of uncertainty. They did not know the outcome of the situation, what would happened to themselves or to their babies. A need for continuous information was expressed, from both of the parents.

*“- There has been a lot of information these days, it is still a bit unclear what is what, and what was said? Then, it is nice to be two to hear the information (looks at her husband). There are so many new impressions all the time. I think it has been so much throughout the whole pregnancy. Still, now afterwards, for both me and her (looks at her daughter).* (Interviewee number 7, 29 years, nulliparous).

The women did not realize why multiple blood samples, clinical examinations and medications were needed. They described a lack of information, and did not experience involvement in medical decisions.

*“- And then they came with medicine, and said take it! But I don´t want to take it, I have to talk to a doctor first. So I said, what´s the plan, should I take medicine all my life? Should I be sent home today? They have to be a little clearer, and not say that all the blood samples looks great. You need a doctor to explain to you why you have to take your medicine”.* (Interviewee number 5, 41 years, nulliparous).

Women hospitalized for several days before the birth appreciated that personnel from the NICU came and informed them about the unit, in case the baby would need to stay there after delivery. Other women and partners were more unprepared for the separation of the new family after the CS, when the mother recovered for a couple of hours at the post-operative unit.

*“The days I was hospitalized before the birth, I received information from the personnel from NICU. They came and informed what to expect if you had a premature baby, how the routines and rooms were organized. So it felt good to know, that it was a room for four and so… (* Interviewee number 9, 29 years, two parous).

## Discussion

The main findings in this study showed that experience of PE is a condition of uncertainty. This phenomenon is characterised in terms of incomprehensible diagnosis message, ambivalent feelings when the unexpected happens, confusing contradictory messages, appreciated support from the midwife, and the need for continuous information. The women described vague symptoms, feeling lack of information and receiving contradictory information from health care professionals, combined with a worrying feeling that the condition quickly can deteriorate and become life threatening for both mother and child.

The results show that it was incomprehensible experiences for nulliparous women to distinguish between normal pregnancy or PE symptoms, and frightening for those with previous experiences or knowledge of PE. The Swedish routine monitoring at the MHC is designed to find serious illnesses among pregnant women, including gestational hypertension and PE. It is important that midwives have a responsiveness to symptoms that can belong to the PE syndrome, to listen, as well as inform and guide the pregnant women regarding further management [30]. The findings in this study shows that women with PE do not always present with the classical symptom, but experience other bothersome symptoms, or show no symptoms at all. Carter et al. [19], made a narrative synthesis of factors that affected women´s help-seeking behaviour regarding PE. They found that there is still a lack of understanding and knowledge of PE, which prevent women from seeking timely and appropriate medical support. The review included studies from several countries, such as Brazil, USA, Bangladesh, and Jamaica. In Jamaica, there was a demarked decline in the incidence of eclampsia six month after women had been offered information via pictorial cards, posters and antenatal education. This led to an increased awareness among women and the health professionals, and made pregnant women more alert to prodromal symptoms, and thereby seek medical advice at the hospital at an earlier time point in pregnancy [31].

This unawareness of PE symptoms can lead to delayed help-seeking, and a deteriorated condition for both mother and child [19]. In fact, this is an important contributing factor to why women in low- and middle-income countries die from this condition [19]. Partly this is also due to a lack of midwives worldwide. World Health Organization (WHO) has calculated that with enough midwives performing family planning and interventions for maternal and newborn health, about 80% of these mother and/or children’s deaths could be prevented [32].

The unawareness of PE symptoms was also found in a study from the UK, where several women expressed a surprise when they suddenly were admitted to hospital due to high blood pressure and proteinuria measured during their routine antenatal clinic appointment [20]. However, women that already had experienced PE were more aware and recognised potential signs and symptoms at an earlier stage and sought care [20]. These findings are in line with one of the women in this study, who previously had lost a baby due to PE in gestational week 25. Furthermore, women who previously experienced PE can be shocked and frightened when diagnosed with PE again, because they understand the life-threatening nature of the disease.

In this study, four out of the nine women experienced central chest pain/pressure when their condition deteriorated, and all of them were delivered by CS. In the descriptions of PE, abdominal pain is often said to be epigastric and/or localized under the right arcus, or upper abdominal pain [7]. Four women in this study placed their hand on the central part of their sternum. They described an intense painful pressure and restricted breathing ability. This description was surprising but mentioned by several of the women.

The women in this study talked about ambivalent feelings when the unexpected happened, like a sudden admission or a conversion to CS. Since PE is unpredictable and can change the natural course of a healthy pregnancy, the unprepared woman may have to be admitted to hospital, have an immediate delivery or emergency CS, and become a mother to a premature baby. This can be a traumatic situation and a heavy burden both physically and emotionally for the mother, and women with these unexpected experiences may have a higher risk of developing PTSD (posttraumatic stress disorder) and postnatal depression [33, 34]. Other studies have concluded that PTSD is more common after traumatic birth experience but that also depends on the individual woman´s coping strategies, her beliefs and social network [35, 36]. Cowan et al. states that it is important that women who experienced severe PE get the opportunity to debrief in the aftermath, preferably with a multidisciplinary team [35].

The women needed to understand and accept their new situation and its consequences. They face both physical and psychological challenges along the way; their own illness, the recovery after a CS, their concerns about becoming a mother in general, and in addition maybe a baby that needs NICU or in the worst case dies. Different coping strategies are described in the literature, depending on the women´s cultural-, beliefs-, and social systems as well as psychological robustness [19, 37, 38]. This knowledge is essential for professionals, as well as partners and family members, to understand and be able to support the woman [39].

A recently published study [17] has shown that women being in a stressful situation like this, are even if they receive information, are not always able to grasp the consequences, and that health care professionals must ensure that the information is repeated and understood [33, 40–42]. Something that also can add to the problem of being able to process information, is when women get severe PE and the HELLP syndrome (Haemolysis Elevated Liver enzymes and Low Platelet count), which has been shown to affect their mental capacity. Both this and other studies investigating women´s experiences of PE show an unmet need for information [16, 17, 42]. The information should preferably be repeated, from the time of diagnosis, throughout pregnancy, before and after childbirth, during the early postpartum period as well as later during the first year to follow up on both mental and physical health.

In this study, seven out of nine women were delivered by emergency CS. This suggests that it is important to inform both the parents that it is more common that women diagnosed with PE are delivered by CS and provide details about the potential family separation at an early stage of their hospitalization. International guidelines recommends vaginal delivery as it is safer and more favourable physiologically [7], yet women affected by PE have a higher risk of delivery by CS [43].

They also need information that prepares them for a potential longer postnatal stay for the condition to stabilize. Harrison et al. [44] concluded that women prefer detailed and realistic information as early as possible, and to take part in their health care decisions. Information consensus and harmonized guidelines between the antenal- and inpatient care, regarding diagnosis, management and follow up plan, is something that reassures the pregnant woman that she is getting adequate care. These benefits are seen when working in well functioning obstetrical teams [45].

The women delivered by emergency CS were all surprised and sad to be separated for several hours from their new-borns and partners, while recovering at the postoperative unit. The fathers who attended the interviews were also surprised, confused and wondered where their wives were taken and when they were coming back. This enlightens the need to also support the woman´s partner, who may have experienced a traumatic situation. Vearland et al. describes the partners experiences as – “becoming a family through reflection on life and death in a context of separation” [46].

In this study the midwives were greatly appreciated for their support and care, and helped the women understand the implications of the diagnosis. However, official information from maternity care providers appeared to be lacking, which led to an increased feeling of uncertainty in many of the women about the situation.

The results show that in order to facilitate early detection and promote a help-seeking behaviour an individual plan needs to be made based on risk factors and previous experiences. Recent studies support these findings, that varied views about management suggests the need for shared decision-making and educational tools [47, 48].

An important clinical implication would be to standardize an antenatal PE education for pregnant women, to be given during antenatal care, preferably combined with web-based training, and mobile phone applications, to enhance further awareness of PE risk factors and symptoms.

### Methodological considerations

When exploring a new research area, the phenomenological approach is especially suited. The approach aims to describe and understand people´s experiences of different phenomenon, without preconceptions and with an opened mind [23, 26, 49]. Using the phenomenological lifeworld approach is a major strength of this study, as it is suitable for gaining a deeper understanding of women’s experiences of a pregnancy complicated with PE. Great care was taken to display the five steps in the analysis process, in order to achieve trustworthiness, to keep one´s mind open through the whole process and to be sensitive to nuances and changes in meaning. The analysis is made as close as possible to the informants own words, and only concentration, not interpretation is made between the different steps. Compte, a French sociologist, meant that instead of imposing a meaning from the outside on the phenomenon, this methodical stance means to wait for the phenomenon to show its meaning to us [50]. The first author was constantly aware of bracketing her pre-understandings, both during the interviews and during the analyses. To increase the credibility, reliability and minimise bias in the results, two researchers (G.A, M.A) interacted by reviewing and giving comments, and critically examine all the steps of the analysis work [49]. Furthermore, all the women were hospitalized at the same maternity unit, and they were all treated according to the same routines and information procedures.

This study poses some methodological limitations. Firstly, this study involved nine women; however, it is not obvious that a larger number of cases would give a different result. The sample size in qualitative studies is valued from the information gained, the power is reflected in how comprehensive the description of the phenomenon is [51]. Furthermore, some fathers attended and gave their inputs in certain questions. This clarified the answers and extended the understanding, by incorporating their view of how PE affected their partners. However, these aspects need to be kept in mind when interpreting the results.

The transferability of qualitative studies is always questionable [40]. A relatively small range of gestation ages were included (range 31–38 + 3 GW). A wider range and/or more severe cases would maybe give different answers. The PE population in the catchment area mainly consists of Scandinavian women (Caucasian ethnicity). The lack of heterogeneity may affect the transferability of the results and may therefore not apply to other settings or ethnic groups. As well known, women from Africa [52] and South America have a higher incidence of PE, and are more prone to develop severe PE and other obstetric complications [9, 52]. Also very young women and women over 40 are more susceptible to severe PE [9]. Therefore, future studies ought to plan for a multi-centre setting, to include women from different ethnicities, with different risk factors, gestation length, and severity to improve the transferability of the results.

Finally, the interviews were mainly performed on the day of discharge, which was not optimal due to disturbing factors such as information from various health care professionals and clinical examinations on both the mothers and their babies. At the same time, it was the time point when they had recovered to a level when they could be dismissed. This may affect how well the phenomenon was narrated.

## Conclusion and implications

This qualitative study reveal a need for improved clinical management. Health care professionals must be aware that women and their partners need detailed, consistent and repeated information about severity and prognosis to diminish the condition of uncertainty, confusion and fearful experience. The clinical implication could be a standardized preeclampsia education for pregnant women early on in the pregnancy, to raise awareness of preeclampsia symptoms. Such educational program needs to be evaluated in an intervention research. Furthermore, there is a need for harmonized guidelines at both the antenatal care and the maternity ward and inpatient care at the hospital. A multi-centre study consisting of a heterogeneous sample of women, and a study exploring the partners experience of unexpected pregnancy and delivery at the PE condition, might give valuable information on how to best support the new family.

## Data Availability

The dataset analysed during the current study is available from the corresponding author on reasonable request.

## Abbreviations

BP: Blood Pressure
dBP: Diastolic Blood Pressure
sBP: Systolic Blood Pressure
BMI: Body Mass Index
CS: Caesarean section
FGR: Fetal Growth Restriction
GH: Gestational Hypertension
HDP: Hypertensive Disorders of Pregnancy
HELLP: Haemolysis Elevated Liver enzymes and Low Platelets
ICU: Intensive Care Unit
IUGR: Intra Uterine Growth Restriction
IVF: In Vitro Fertilisation
ISSHP: International Society for the Study of Hypertension in Pregnancy
NICE: National Institute for Health and Care Excellence
NICU: Neonatal Intensive Care Unit
PE: Preeclampsia
SGA: Small for Gestation Age
GW: Gestation Weeks
MHC: Maternal Health Care
